# Integrated management enhances crop physiology and final yield in maize intercropped with blackgram in semiarid South Asia

**DOI:** 10.3389/fpls.2022.975569

**Published:** 2022-09-23

**Authors:** T. Varatharajan, Anchal Dass, Anil K. Choudhary, S. Sudhishri, V. Pooniya, T. K. Das, G. A. Rajanna, Shiv Prasad, Karivaradharajan Swarnalakshmi, M. N. Harish, Shiva Dhar, Raj Singh, Rishi Raj, Kavita Kumari, Arjun Singh, K. S. Sachin, Pramod Kumar

**Affiliations:** ^1^Indian Agricultural Research Institute, Indian Council of Agricultural Research, New Delhi, India; ^2^Central Potato Research Institute, Indian Council of Agricultural Research, Shimla, India; ^3^Directorate of Groundnut Research, Indian Council of Agricultural Research, Ananthapur, India; ^4^Farm Science Centre, Indian Institute of Horticultural Research, Indian Council of Agricultural Research, Gonikoppal, India; ^5^National Research Centre for Banana, Indian Council of Agricultural Research, Tiruchirappalli, India

**Keywords:** integrated crop management, blackgram intercropping, conservation agriculture, cropping systems, dry matter partitioning, photosynthetic rate

## Abstract

Photosynthesis, crop health and dry matter partitioning are among the most important factors influencing crop productivity and quality. Identifying variation in these parameters may help discover the plausible causes for crop productivity differences under various management practices and cropping systems. Thus, a 2-year (2019–2020) study was undertaken to investigate how far the integrated crop management (ICM) modules and cropping systems affect maize physiology, photosynthetic characteristics, crop vigour and productivity in a holistic manner. The treatments included nine main-plot ICM treatments [ICM_1_ to ICM_4_ – conventional tillage (CT)-based; ICM_5_ to ICM_8_ – conservation agriculture (CA)-based; ICM_9_ – organic agriculture (OA)-based] and two cropping systems, *viz*., maize–wheat and maize + blackgram–wheat in subplots. The CA-based ICM module, ICM_7_ resulted in significant (*p* < 0.05) improvements in the physiological parameters, *viz*., photosynthetic rate (42.56 μ mol CO_2_ m^–2^ sec^–1^), transpiration rate (9.88 m mol H_2_O m^–2^ sec^–1^) and net assimilation rate (NAR) (2.81 mg cm^–2^ day^–1^), crop vigour [NDVI (0.78), chlorophyll content (53.0)], dry matter partitioning toward grain and finally increased maize crop productivity (6.66 t ha^–1^) by 13.4–14.2 and 27.3–28.0% over CT- and OA-based modules. For maize equivalent grain yield (MEGY), the ICM modules followed the trend as ICM_7_ > ICM_8_ > ICM_5_ > ICM_6_ > ICM_3_ > ICM_4_ > ICM_1_ > ICM_2_ > ICM_9_. Multivariate and PCA analyses also revealed a positive correlation between physiological parameters, barring NAR and both grain and stover yields. Our study proposes an explanation for improved productivity of blackgram-intercropped maize under CA-based ICM management through significant improvements in physiological and photosynthetic characteristics and crop vigour. Overall, the CA-based ICM module ICM_7_ coupled with the maize + blackgram intercropping system could be suggested for wider adoption to enhance the maize production in semiarid regions of India and similar agroecologies across the globe.

## Introduction

Photosynthesis, crop health and the accumulation and distribution of dry matter among different plant parts determine the level of crop yield (Rana et al., 2014). Hence, understanding the crop physiological and crop growth mechanism behind yield increase may help in the extrapolation of newly developed crop management technologies to other crops with judicious use of external inputs and farm resources. It has become more essential under the aegis of the UN’s Sustainable Development Goals (SDGs) to achieve zero hunger, good health and well-being, clean environment and resources and clean production with appropriate climate action (Harish et al., 2022). As per an estimate, the world population will increase from the current 7.9 to 9.7 billion by 2050, hence, requiring ∼70–100% increase in the production of major cereal crops (Guo Y. et al., 2021). Alike, the rice–wheat cropping system (RWCS), being a dominant cropping system in the South-Asian Indo-Gangetic plains region (IGPR), hinges food security of resource-poor and economically weak population (Choudhary et al., 2021, 2022). But there are several issues in RWCS in the South-Asian IGPR as indiscriminate use of chemical fertilisers, land degradation (salinisation/alkalisation), low resource-use efficiencies, water table decline and higher associated cost and energy in water extraction (Kumar et al., 2021, 2022; Harish et al., 2022), besides substantial weed management costs and the emergence of weed resistance (Dass et al., 2017; Choudhary et al., 2021), severe incidence of pests, diseases and minor pest resurgence, climatic vulnerabilities like heat/cold waves, terminal drought stress and other numerous production vulnerabilities that collectively threaten the sustainability of RWCS in IGPR (Paul et al., 2014; Bhatt et al., 2016; Pooniya et al., 2018, 2022). To overcome these vulnerabilities, one strategy could be to replace rice with maize, legumes and other underutilised crops that require less water and other resources (Choudhary et al., 2018, 2020; Harish et al., 2021, 2022; Singh et al., 2021, 2022a; Pooniya et al., 2022); and hence, diversify the system for better productivity, profitability and resource use.

With the advent of several high-yielding hybrids and bio-fortified cultivars, maize has become a highly competitive crop in replacing the RWCS in view of better farm productivity, profitability, sustainability and nutritional security (Yadav et al., 2015). Moreover, maize has a wide adaptability to diverse agroclimatic conditions making it a potential alternative (Sharma and Dass, 2012). However, there exists a yawning gap between developed countries and India for maize productivity (Yadav et al., 2015). Therefore, scientists in India are working hard to raise its productivity to the global level. Among important options, legume inclusion makes the cropping systems more profitable and resilient with improved soil health (Dass and Sudhishri, 2010; Weil and Brady, 2017; Choudhary et al., 2020). Likewise, legume as an intercrop could be a low-input strategy in agriculture for improving the food, nutritional, economical and environmental security of the small and marginal farm families (Maitra et al., 2021), besides reducing the risks of crop failure, improving the system sustainability, reducing the soil erosion and preventing nutrient leaching losses (Dass and Sudhishri, 2010; Nyawade et al., 2019). In order to improve the resource-use efficiency (RUE) and to facilitate the intercropping advantages like niche complementarity and enhanced system productivity, the selection of appropriate intercrop combinations with synergistic effects is highly essential (Choudhary et al., 2020). There are several reports on the use of blackgram as an intercrop in maize under conventional cultivation methods. In cereal forages, legume intercropping increases dry matter, crude protein and lowers neutral detergent fibre (Zhang et al., 2015). Maize intercropped with legumes, such as alfalfa, soybean, fababean, greengram, and blackgram, improves the RUE, N and P uptake, system productivity and net return compared to sole maize cropping by judicious usage of space and sunlight, resources, weed smothering, increased residue decomposition rate, rhizosphere microbial community and legume nodule activity (Sangakkara, 1994; Li et al., 2003; Zhang et al., 2011; Sun et al., 2014; Singh et al., 2021, 2022a,2022b; Tripathi et al., 2021). However, there is no study on blackgram intercropping in maize under different integrated crop management (ICM) modules. Hence, this prompted us to study how the blackgram intercropping in maize changes the growth, physiology and productivity of maize crop mediated by various ICM modules.

The Food and Agriculture Organization (FAO) has recently adopted the ICM as a holistic site-specific approach to crop husbandry that combines the sustainable tillage and land configuration practices and the integrated nutrient, weed, water and pest management practices to deliver the most efficient and safe farm output with long-term benefits while conserving/enhancing the natural resources (Varatharajan et al., 2019a; Choudhary et al., 2020; Biswakarma et al., 2021). Globally, ICM has gained importance because of numerous crop production and resource constraints associated with major cropping systems, *viz*., rice–wheat, maize–wheat, pigeonpea–wheat and soybean–wheat, etc., in India *per se*. The application of ICM techniques helps in raising RUE and system productivity (Choudhary et al., 2020). Earlier reports in major cropping systems of India like RWCS, maize–wheat cropping system (MWCS), soybean–wheat under ICM indicated that CA-based treatments recorded higher crop yields due to improved crop physiological and photosynthetic characteristics, modulation of microclimate and resilience to environmental stresses, enhanced soil fertility and favourable soil microbiome (Choudhary et al., 2018, 2020; Varatharajan et al., 2019a; Singh et al., 2020, 2021, 2022a; Biswakarma et al., 2021; Pooniya et al., 2022). Our extensive literature review revealed that the majority of earlier studies focused on individual components of crop management, i.e., crop establishment, tillage, nutrient, weed, water, energy management, etc.; meagre information is available on adopting the ICM technology by integrating all input- and production-related factors in the crops. The information pertaining to the combination/interaction effect of ICM and cropping system (sole vs. intercrop) in the MWCS under different tillage, crop establishment patterns, residue retention, nutrient management, etc., under intercropping is also inadequate. Therefore, the current study was designed for investigating this important research issue to bridge the knowledge gap and to decode the intrinsic interrelationship among leaf and photosynthetic parameters, dry matter partitioning (DMP) and maize productivity vis-à-vis resilience to environmental stresses. All this is expected to provide insights into understanding the mechanism of yield advantages under ICM in general and intercropping combinations of maize grown under MWCS of semiarid IGPR in South Asia. The overall objective of the study was to understand whether and how far do the ICM modules (tillage, fertiliser, irrigation and weed management, applied in combination) and cropping systems affect maize physiology, photosynthetic characteristics, crop vigour and productivity in a holistic manner.

## Materials and methods

### Experimental site, climate and soil

**FIGURE 1:**
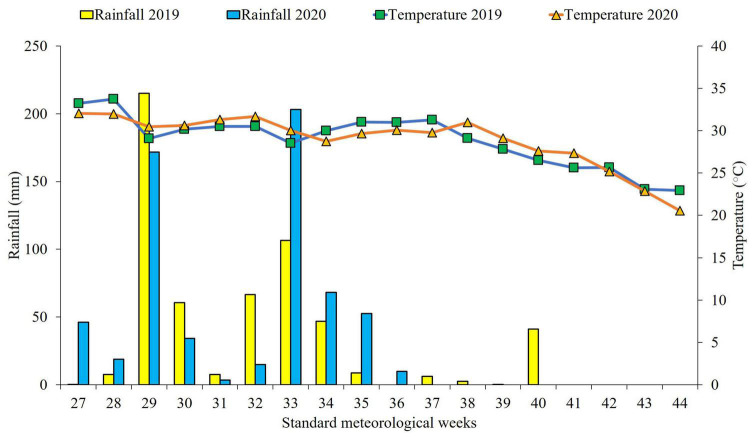
Weekly rainfall and mean temperature during crop growing seasons (2019–2020). [Source: Agro-meteorological Observatory, Division of Agricultural Physics, ICAR-IARI, New Delhi].

A field experiment was conducted in two consecutive cropping cycles (*kharif* season) during 2019 and 2020 in the research farm of ICAR-Indian Agricultural Research Institute, New Delhi [Latitude 28° 63’ N; Longitude 77° 15’ E; Altitude 228.6 m], under Indo-Gangetic plains region. The experimental site is located in a semiarid region with *Typic Ustochrepts* sandy loam alluvial soil with subtropical climate having hot, dry summers and cold winters. Mean weather parameters recorded in the two respective cropping seasons are presented in Table 1 and Figure 1. The experimental soil had a pH 8.23, soil organic carbon (SOC) 0.48% and available nitrogen (N), phosphorus (P) and potassium (K) contents of 194.1, 14.8 and 303.4 kg ha^–1^, respectively, for 0–15 cm soil layer as estimated using standard protocols (Rana et al., 2014).

**TABLE 1:** Mean agro-meteorological parameters recorded during the study (2019–20 and 2020–21).

Meteorological parameters	2019–2020 Kharif	2020–2021 Kharif
Max temp. (^°^C)	33.6	34.5
Min temp. (^°^C)	24.2	23.3
Total rainfall (mm)	569.5	622.8
Relative humidity (%) (M)	87.3	85.1
Relative humidity (%) (E)	63	56.3
Sunshine hour (h day^–1^)	4.0	5.9
Evaporation (mm day^–1^)	4.1	4.7
Wind speed (km h^–1^)	3.6	4.3

In the present study, Kharif season denotes the period between 2nd July and 4th November.

### Experimental design

In the current study, nine main-plot treatments and two subplot treatments, all replicated thrice, were evaluated using a split-plot design. Main plots (42.0 m^2^ each) were assigned to nine ICM modules [ICM_1_ to ICM_4_ – conventional tillage (CT)-based; ICM_5_ to ICM_8_ – conservation agriculture (CA)-based; ICM_9_ – organic agriculture (OA)], while subplots (18.9 m^2^ each) were allotted to two cropping systems: (1) maize–wheat and (2) maize + blackgram–wheat. There was a total of 54 experimental units. Prior to the experiment, the field was under soybean–wheat cropping system for 4 years and ICM with the same layout. No synthetic fertilizer and crop protection chemical were used under OA (ICM_9_); only farmyard manure (FYM) was used as a nutrient source, with crop protection provided by botanicals, such as neem seed kernel extract. The detailed treatment description is presented in Table 2.

**TABLE 2:** Details of nine ICM modules (main plot) followed in maize/maize + blackgram system.

ICM modules	ICM_1_	ICM_2_	ICM_3_	ICM_4_	ICM_5_	ICM_6_	ICM_7_	ICM_8_	ICM_9_
Tillage	CT	CT	CT	CT	CA	CA	CA	CA	CT
Planting pattern	FB	FB	RB	RB	FB	FB	PRB	PRB	FB
Wheat CRR	–	–	–	–	+	+	+	+	+
Nutrient management	100% RDF	75% RDF	100% RDF	75% RDF	100% RDF	75% RDF	100% RDF	75% RDF	FYM 15 t ha^–1^
NPK biofertiliser + AM fungi	–	+	–	+	–	+	–	+	+
Irrigation depth irrigation^–1^	60 mm	60 mm	45 mm	45 mm	60 mm	60 mm	45 mm	45 mm	60 mm
Weed management	Pendi and Tembo	Pendi and Tembo	Pendi and Tembo	Pendi and Tembo	Glypho, Pendi and Tembo	Glypho, Pendi and Tembo	Glypho, Pendi and Tembo	Glypho, Pendi and Tembo	Hand weeding and its mulching

CT, conventional tillage; ZT, zero tillage; FB, flatbed; PRB, permanently raised bed; CRR, crop residue retention (3 t ha-); RDF, recommended dose of fertiliser; Glypho, glyphosate pre-plant application 1 kg a.i. ha^–1^; Pendi, pendimethalin pre-emergence 1 kg a.i. ha^–1^; Tembo, tembotrione post-emergence 0.11 kg a.i. ha^–1^. Integrated pest and disease management was adopted irrespective of treatments. In blackgram-intercropped maize plots, hand weeding was adopted instead of tembotrione post-emergence application.

### Crop management

The experimental field was prepared using a tractor-drawn double-disc MB plough followed by two cultivator-cum-planker operations under CT. Whereas in CA, zero-tillage (ZT) plots were prepared by glyphosate spray (1 kg a.i. ha^–1^) 15 days before sowing. Maize (*Zea mays*) cultivar ‘PMH-1’ was sown at 70 × 20 cm spacing using 20 kg seed ha^–1^. The additive series intercropping technique was adopted maintaining 100% population of maize (base crop) in blackgram-intercropped subplots. Blackgram (*Vigna mungo*) cultivar ‘Pant-U-30’ was sown at a seed rate of 10 kg ha^–1^ to establish two rows of blackgram (at 35 × 10 cm spacing) between every two rows of maize. Both maize and maize + blackgram were sown at the same time (15th and 13th July in 2019 and 2020, respectively), while the blackgram was harvested earlier to maize in both years (30th and 27th September in 2019 and 2020, respectively). The maize was harvested in 25th and 22th October in 2019 and 2020, respectively. The wheat crop under cropping system was cultivated in the same year, and the period between wheat harvest and maize sowing was 3 months. The experimental treatments were repeated exactly in the same plots for both years to know the cumulative/additive effect of legume intercropping in the cropping systems. Three crop establishment patterns were followed among different ICM modules: (i) flatbed (FB), (ii) raised bed (RB) and (iii) permanent raised bed (PRB). The RB/PRB were prepared with a width of 70 cm. Crop residues from the preceding wheat crop were retained at 3 t ha^–1^ under CA-based modules (ICM_5_–ICM_8_) and OA module (ICM_9_). Three nutrient management schedules were followed in different ICM modules, *viz*., (i) 100% recommended dose of fertiliser (150:80:60 kg N:P_2_O_5_:K_2_O ha^–1^) (100% RDF), (ii) 75% RDF (112.5:60:45 kg N:P_2_O_5_:K_2_O ha^–1^) + NPK biofertiliser + arbuscular mycorrhizal (AM) fungi (75% RDF + NPK-*bf* + AMF) both in CT- and CA-based modules (ICM_1_–ICM_8_) and while (iii) FYM at 15 t ha^–1^ + NPK-bf + AM fungi (FYM_15_ + NPK-*bf* + AMF) in OA module (ICM_9_). Irrespective of treatments, the entire amounts of fertiliser P and K were basally applied, while N was supplied in three equal splits (at basal, knee-high and tasselling stage in maize). Although the number of irrigations was common for all treatments (3 and 4 irrigations were scheduled in 2019 and 2020, respectively), the depth of water applied in each irrigation varied with crop establishment patterns, i.e., (i) 60 mm in FB and (ii) 45 mm in RB/PRB (Table 2), measured and applied accurately using portable water meter installed in field channels. Blackgram (intercrop) was harvested 30 days earlier than maize harvest in both study years. The crop from the net-plot area (chosen at centre of the plot, by excluding border rows) was harvested manually using sickles and then sun-dried at threshing floor. Tractor-operated maize dehusker-cum-sheller was used to separate grains from corncob, while blackgram from intercropped plots was harvested, threshed and cleaned manually. The grains were sun-dried to 14% seed moisture, cleaned to record grain and stover yields of both maize and blackgram (Rana et al., 2014). Border rows were excluded from all samplings/recording observations to avoid any over/underestimation of parameters involved.

### Leaf and photosynthetic characteristics and net assimilation rate

Leaf photosynthetic characteristics were measured using an infrared gas analyser (IRGA) LI-6400 XT that is a portable photosynthetic system (Li-COR, Lincoln, NE, USA), during the flowering stage on a clear sunny day between 9.00 and 11.00 h. The airflow rate through the chamber was 500 μmol s^–1^, the CO_2_ concentration was the ambient one, and relative humidity was 70–75%. Three plants per plot were randomly selected, and the measurements, *viz.*, (i) net photosynthetic rate (P_*n*_) – amount of CO_2_ consumed by leaves per unit area per unit time (μ mol CO_2_ m^–2^ sec^–1^), (ii) transpiration rate (T_*r*_) – the amount of water consumed by leaves per unit area per unit time (m mol H_2_O m^–2^ sec^–1^), (iii) stomatal conductance (G_*s*_) – gas exchange of stomata per unit area per unit time (mol H_2_O m^–2^ sec^–1^), were taken from its uppermost fully expanded leaf. The transpiration efficiency (T_*E*_) – the amount of CO_2_ assimilation per unit mass of water transpired [μ mol CO_2_ (m mol H_2_O)^–1^] – was computed using the following formula:


$$T_{E}=\frac{P_{n}}{T_{r}}$$

The net assimilation rate (NAR) (Williams, 1946) that represents the increase in dry weight per unit leaf area per unit time was calculated at 30-day intervals, starting from 30 DAS and continuing till 90 DAS.

### Normalised difference vegetation index

Normalised difference vegetation index (NDVI) is a dimensionless vegetation index and an indicator of greenness vis-à-vis vigour of vegetation, used to evaluate the density of greenness and crop health. NDVI values were measured using a handheld crop sensor Trimble GreenSeeker^®^ (Trimble, Sunnyvale, CA, USA). The instrument’s sensor emits red and infrared light and then measures the amount of light reflected from each segment. The average NDVI value of the respective plot was recorded from the sensor at 50 cm height above the crop canopy at 30-day intervals.

### Leaf chlorophyll content (soil plant analysis development)

Chlorophyll content (SPAD) or greenness of the plant was measured using Konica-Minolta SPAD-502 chlorophyll meter (Minolta, Osaka, Japan). Ten plants per plot were randomly selected and tagged to measure the SPAD value at 30-day intervals. From each plant, three readings were recorded per leaf, and the mean was calculated to get the SPAD value of the respective treatment.

### Plant dry matter partitioning

Five representative plants per plot were randomly selected for measuring DMP during the flowering and harvest stage. To record DMP of respective plant parts, i.e., leaves, stem, husk, corncob and kernels per plant were separated and shade-dried followed by oven drying (65 ± 2°C), weighed on achieving the constant weight and recorded in g plant^–1^ for respective plant part (Rana et al., 2014).

### Leaf area index and leaf growth parameters

Five plants per plot were randomly selected for measuring leaf area at 30-day intervals using LI-3100C leaf area meter (Li-COR, Lincoln, NE, USA) as cm^2^ plant^–1^, and the leaf area index (LAI) was calculated (Rana et al., 2014). Based on plant dry matter and leaf area observations recorded, the following leaf growth indices were calculated using the formulas given below: leaf area ratio (Radford, 1967), leaf weight ratio, specific leaf area (Kvet et al., 1971), specific leaf weight (Pearce et al., 1968).


$$\mathrm{Leaf}\,\mathrm{area}\,\mathrm{index}=\,\frac{\mathrm{Leaf}\,\mathrm{area}\,\mathrm{per}\,\mathrm{plant}\,\left({\text{cm}}^{2}\right)}{\mathrm{Ground}\,\mathrm{area}\,\mathrm{occupied}\,\left({\text{cm}}^{2}\right)}$$

$$\mathrm{Leaf}\,\mathrm{area}\,\mathrm{ratio}\,\left({\text{cm}}^{2}\,g^{-1}\right)=\,\frac{\mathrm{Leaf}\,\mathrm{area}\,\left({\text{cm}}^{2}\right)\,}{\mathrm{Plant}\,\mathrm{dry}\,\mathrm{weight}\,\left(\text{g}\right)}$$

$$\mathrm{Leaf}\,\mathrm{weight}\,\mathrm{ratio}=\,\frac{\mathrm{Leaf}\,\mathrm{dry}\,\mathrm{weight}\,\left(\text{g}\right)}{\mathrm{Plant}\,\mathrm{dry}\,\mathrm{weight}\,\left(\text{g}\right)}$$

$$\mathrm{Specific}\,\mathrm{leaf}\,\mathrm{area}\,\left({\text{cm}}^{-2}\,g^{-1}\right)=\,\frac{\mathrm{Leaf}\,\mathrm{area}\,\left({\text{cm}}^{2}\right)\,}{\mathrm{Leaf}\,\mathrm{dry}\,\mathrm{weight}\,\left(\text{g}\right)}$$

$$\mathrm{Specific}\,\mathrm{leaf}\,\mathrm{weight}\,\left(g\,{\mathrm{cm}}^{-2}\right)=\,\frac{\mathrm{Leaf}\,\mathrm{dry}\,\mathrm{weight}\,\left(\text{g}\right)\,}{\mathrm{Leaf}\,\mathrm{area}\,\left({\text{cm}}^{2}\right)}$$

### Days taken to different phenological stages

Ten plants per plot were selected randomly and tagged to observe number of days taken (after sowing) for the attainment of different phenological growth stages, *viz*., days taken to 50% tasselling and days taken to 50% silking.

### Crop productivity

#### Maize equivalent grain yield

Maize equivalent grain yield (MEGY) denotes the financial sum of maize grain yield and MEGY of blackgram. Thus, MEGY of blackgram was determined using the following expression:


$$M\,E\,G\,Y\,o\,f\,b\,l\,a\,c\,k\,g\,r\,a\,m\,\;\,\left(t\,h\,a^{-1}\right)$$

=B⁢l⁢a⁢c⁢k⁢g⁢r⁢a⁢m⁢g⁢r⁢a⁢i⁢n⁢y⁢i⁢e⁢l⁢d⁢(t⁢h⁢a-1)×P⁢r⁢i⁢c⁢e⁢o⁢f⁢ 1⁢t⁢b⁢l⁢a⁢c⁢k⁢g⁢r⁢a⁢m⁢g⁢r⁢a⁢i⁢n⁢P⁢r⁢i⁢c⁢e⁢o⁢f⁢ 1⁢t⁢o⁢f⁢m⁢a⁢i⁢z⁢e⁢g⁢r⁢a⁢i⁢n

The minimum support price (decided by the Government of India) prevailing during the cropping season was used in above calculations, i.e., for maize INR 17600 (USD 235) and INR 18500 (USD 247) t^–1^ during 2019 and 2020, respectively, and similarly for blackgram INR 57000 (USD 760) and INR 60000 (USD 800) t^–1^ during 2019 and 2020, respectively.

#### Maize equivalent stover yield

Maize equivalent stover yield (MESY) (combined stover yield) denotes the sum of maize stover yield and MESY of blackgram. Thus, MESY of blackgram was calculated as per the following formula:


$$M\,E\,S\,Y\,o\,f\,b\,l\,a\,c\,k\,g\,r\,a\,m\,\;\,\left(t\,h\,a^{-1}\right)$$

=B⁢l⁢a⁢c⁢k⁢g⁢r⁢a⁢m⁢s⁢t⁢o⁢v⁢e⁢r⁢y⁢i⁢e⁢l⁢d⁢(t⁢h⁢a-1)×P⁢r⁢i⁢c⁢e⁢o⁢f⁢ 1⁢t⁢b⁢l⁢a⁢c⁢k⁢g⁢r⁢a⁢m⁢s⁢t⁢o⁢v⁢e⁢r⁢P⁢r⁢i⁢c⁢e⁢o⁢f⁢ 1⁢t⁢o⁢f⁢m⁢a⁢i⁢z⁢e⁢s⁢t⁢o⁢v⁢e⁢r

The stover prices were determined based on the prevailing local market prices. Both maize and blackgram stover were priced at INR 3000 (USD 40) t^–1^ during both the years.

### Statistical analysis

The differences between the treatments were statistically analysed by ANOVA technique using JMP^®^ from SAS. The significant difference between the mean values of treatments was determined by using the least significant difference (*p* < 0.05) and indicated by different letters. Standard error of mean values (SEm±) was provided in all appropriate places. Multivariate analysis (MVA) and principal component analysis (PCA) were carried out using JMP^®^ from SAS to decipher the association between physiological parameters, crop vigour, plant growth, MEGY and MESY.

## Results

### Photosynthetic characteristics

Irrespective of the ICM treatments, net photosynthetic rate (P_n_) values ranged from 34.3 to 42.9 μ mol CO_2_ m^–2^ sec^–1^ during the flowering stage for maize (Table 3). There was a significant (*p* < 0.05) difference between CT and CA, as well as between CA- and OA-based ICM modules. However, no significant difference existed between 100% RDF- and 75% RDF + NPK-bf + AMF-applied modules in any study years. CA-based ICM_7_ module [ZT + PRB + wheat crop residue retention (3 t ha^–1^) + 100% RDF + glyphosate-PP fb pendimethalin-PE fb tembotrione-POE + 3 irrigations (45 mm depth each) + need-based integrated crop protection] showed 19.3 and 14.3% enhancement in P_n_ over OA- and CT-based modules. Inclusion of blackgram as an intercrop influenced P_n_ in maize significantly (*p* < 0.05); intercropped maize exhibited 4.5% higher P_n_ than sole maize. The transpiration rate (T_r_) values ranged between 9.02 and 9.92 m mol H_2_O m^–2^ sec^–1^. There was a significant (*p* < 0.05) difference between CA and OA, while T_r_ in CT and CA stood alike. The use of 100% RDF and 75% RDF + NPK-*bf* + AMF nutrient management did not differ significantly (*p* < 0.05) from each other. CA-based ICM_7_ module recorded 7.1 and 6.2% higher T_r_ than OA- and CT-based ICM modules, and the differences were significant statistically. Blackgram intercropping improved the Tr in maize by ∼2% over sole maize (Table 3).

**TABLE 3:** Effect of ICM modules and cropping system on net photosynthetic rate (Pn), transpiration rate (T_r_), stomatal conductance (G_s_), transpiration efficiency (T_E_) at flowering stage and net assimilation rate (NAR) at 30-day intervals in maize*^a^*.

Treatments	P_n_ (μ mol CO_2_ m^–2^sec^–1^)		T_r_ (m mol H_2_O m^–2^ sec^–1^)		G_s_ (mol H_2_O m^–2^ sec^–1^)		T_E_ [μ mol CO_2_ (m mol H_2_O)^–1^]		NAR (mg cm^–2^ day^–1^)					
	
									0–30 DAS		30–60 DAS		60–90 DAS	
	2019	2020	2019	2020	2019	2020	2019	2020	2019	2020	2019	2020	2019	2020
*Integrated crop management (ICM)*														
ICM_1_	36.41^de^	36.53^cd^	9.11^bc^	9.15^b^	0.38^def^	0.39^cde^	3.99^cde^	3.99^bcd^	2.49^bc^	2.48^c^	0.41	0.41	0.57^b^	0.56^ab^
ICM_2_	35.22^e^	35.47^d^	9.08^bc^	9.11^b^	0.37^ef^	0.38^de^	3.88^de^	3.89^cd^	2.40^c^	2.38^c^	0.43	0.44	0.58^b^	0.59^a^
ICM_3_	38.69^cd^	38.95^bc^	9.53^abc^	9.57^ab^	0.40^bcd^	0.41^abcd^	4.06^bcd^	4.07^abc^	2.50^bc^	2.56^bc^	0.41	0.39	0.53^c^	0.52^bc^
ICM_4_	38.32^cd^	38.62^bc^	9.48^abc^	9.50^ab^	0.39^cde^	0.40^bcde^	4.05^bcd^	4.07^abc^	2.49^bc^	2.49^c^	0.41	0.40	0.52^c^	0.51^bc^
ICM_5_	40.18^abc^	40.53^ab^	9.81^a^	9.85^a^	0.41^abc^	0.42^abc^	4.09^abcd^	4.12^abc^	2.66^ab^	2.69^ab^	0.41	0.40	0.50^cd^	0.50^c^
ICM_6_	39.58^bc^	39.85^b^	9.63^ab^	9.61^ab^	0.40^abc^	0.41^abc^	4.11^abc^	4.15^ab^	2.54^bc^	2.54^bc^	0.43	0.43	0.52^cd^	0.51^c^
ICM_7_	42.25^a^	42.86^a^	9.84^a^	9.92^a^	0.41^ab^	0.42^ab^	4.29^a^	4.32^a^	2.84^a^	2.77^a^	0.39	0.39	0.50^cd^	0.49^c^
ICM_8_	41.42^ab^	41.44^ab^	9.72^a^	9.74^a^	0.42^a^	0.43^a^	4.26^ab^	4.25^a^	2.83^a^	2.76^a^	0.41	0.40	0.48^d^	0.48^c^
ICM_9_	34.28^e^	34.42^d^	9.02^c^	9.06^b^	0.37^f^	0.37^e^	3.81^e^	3.81^d^	2.47^bc^	2.44^c^	0.43	0.44	0.64^a^	0.57^a^
SEm ±	0.83	0.96	0.19	0.19	0.006	0.009	0.08	0.08	0.07	0.06	0.01	0.01	0.01	0.02
LSD (0.05)	2.50	2.89	0.56	0.56	0.02	0.03	0.23	0.25	0.20	0.19	NS	NS	0.04	0.05
*Cropping systems (CS)*														
M–W	37.61^b^	37.85^b^	9.39^b^	9.41^b^	0.40	0.40	4.00^b^	4.02^b^	2.53^b^	2.54^b^	0.42^a^	0.42^a^	0.55^a^	0.53^a^
M + B–W	39.36^a^	39.63^a^	9.55^a^	9.59^a^	0.39	0.40	4.12^a^	4.13^a^	2.62^a^	2.59^a^	0.41^b^	0.40^b^	0.53^b^	0.52^b^
SEm ±	0.35	0.30	0.04	0.05	0.00	0.00	0.03	0.02	0.01	0.01	0.003	0.002	0.005	0.004
LSD (0.05)	1.05	0.90	0.12	0.15	NS	NS	0.09	0.06	0.04	0.03	0.01	0.01	0.01	0.01
ICM × CS	NS	NS	NS	NS	NS	NS	NS	NS	S	S	NS	NS	NS	NS

For detailed description of ICM, refer Table 2; maize–wheat cropping system (M–W); maize + blackgram–wheat cropping system (M + B–W).

^*a*^Values with different superscript letters in a column are significantly (*p* < 0.05) different.

The stomatal conductance (G_s_) values ranged from 0.37 to 0.43 mol H_2_O m^–2^ sec^–1^ during the flowering stage; CT, CA, and OA-based ICM modules exhibited significant (*p* < 0.05) differences in G_s_ during both years. The G_s_ was comparable between 100% RDF- and 75% RDF + NPK-*bf* + AMF-applied modules. However, the CA-based ICM_8_ module recorded ∼12.9 and 11.8% higher G_s_ than OA- and CT-based modules, respectively. Blackgram intercropping did not show any significant effect on G_s_ in maize. Transpiration efficiency (T_E_) values ranged between 3.81 and 4.32 μ mol CO_2_ (m mol H_2_O)^–1^. CA-based module ICM_7_ recorded significantly (*p* < 0.05) higher T_E_, which indicates 4.29–4.32 μ mol CO_2_ is fixed for every m mol H_2_O transpired. Blackgram intercropping (*p* < 0.05) caused significant improvement (∼3% increase) in T_E_ of maize over sole maize. Further, ICM_9_, the OA-based ICM module, stood at the bottom level for all photosynthetic characteristics studied (Table 3). The ICM and CS interaction was non-significant for photosynthetic characteristics (P_n_, T_r_, G_s_, and T_E_) of maize during both years.

### Net assimilation rate

In general, the NAR ranged from 0.39 to 2.38 mg cm^–1^ day^–1^. Initially (0–30 DAS), NAR was high and later showed a falling trend (Table 3). Crop establishment techniques like FB, RB/PRB did not vary significantly in both CT- and CA-based ICM modules. Similarly, the nutrient management option 75% RDF + NPK-*bf* + AMF stood at par with 100% RDF-applied ICM modules in both CT- and CA-based ICM modules. But, among tillage options, the CA-PRB ICM module (ICM_7_ and ICM_8_) exhibited a significant advantage over CT-based ICM modules (ICM_1_ to ICM_4_) by assimilating more dry matter per unit leaf area per unit time. During the initial stages of growth (0–30 and 30–60 DAS), CA-based ICM modules exhibited higher NAR; however, during later growth stages (60–90 DAS), CT- and OA-based ICM modules showed significantly (*p* < 0.05) higher NAR. The OA-based ICM_9_ showed a low assimilation rate during early stages and significantly higher assimilation rate than CA-based ICM modules in later stages of growth. During 0–30 DAS, the CA-based ICM_7_ assimilated 2.84 and 2.77 mg of dry matter per cm^2^ of leaf area per day, which was 11.4 and 12.5% higher than CT-FB- and OA-based ICM modules. ICM modules did not show significant variation in NAR during 30–60 DAS. But during 60–90 DAS, OA-based ICM_9_ had significantly (*p* < 0.05) higher NAR, 0.64 and 0.57 mg dry matter per cm^2^ of leaf area per day during 2019 and 2020, respectively. Irrespective of the measurement interval, blackgram intercropping significantly altered the NAR in maize. Intercropped maize had significantly (*p* < 0.05) higher (1.9–3.4%) net assimilation though only for 0–30 DAS; at later stages, intercropped maize recorded lower. For the early growth stage (0–30 DAS) only, the ICM × cropping system (CS) interaction emerged significant (*p* < 0.05); both CA-based PRB modules (ICM_7_ and ICM_8_) had a comparable impact, although CT and OA depicted a much stronger interaction effect of blackgram intercropping than CA modules.

### Normalised difference vegetation index

The Normalised Difference Vegetation Index (NDVI) ranged between 0.31 and 0.78, initially increased to the peak value at 60 DAS and then tapered gradually with the progression of plant growth (Table 4). NDVI varied significantly (*p* < 0.05) among CT, CA and OA-based modules. Various tillage, crop establishment techniques, nutrient management options and residue retention had a significant (*p* < 0.05) effect on maize NDVI. Irrespective of other variables, CA-based modules recorded higher NDVI than CT and OA modules. CA-PRB modules registered 24.1 and 35.2% greater NDVI values than CT-FB- and OA-based modules. At 30 DAS, all 100% RDF-applied modules recorded significantly higher NDVI over 75% RDF + NPK-bf + AMF-applied modules. Blackgram intercropping significantly (*p* < 0.05) improved the NDVI at 30 and 60 DAS, the increase being 14.7 and 3.3%, respectively, over sole maize. The OA ICM_9_ recorded significantly lower NDVI at all growth stages.

**TABLE 4:** Effect of different ICM modules and cropping system on Normalised Difference Vegetation Index (NDVI) and chlorophyll content (SPAD) in maize at 30-days interval^a^.

Treatments	NDVI						Chlorophyll content (SPAD)					
	30 DAS		60 DAS		90 DAS		30 DAS		60 DAS		90 DAS	
	2019	2020	2019	2020	2019	2020	2019	2020	2019	2020	2019	2020
*Integrated crop management (ICM)*												
ICM_1_	0.37^ef^	0.38^cd^	0.74^abc^	0.75^abc^	0.46^d^	0.48^cd^	38.05^de^	39.55^d^	48.08^cd^	48.53^cd^	43.68^cd^	43.99^c^
ICM_2_	0.35^f^	0.37^d^	0.72^cd^	0.73^cd^	0.45^d^	0.47^cd^	36.02^ef^	37.23^e^	47.75^cd^	48.10^cd^	42.86^d^	43.11^c^
ICM_3_	0.40^d^	0.39^cd^	0.75^abc^	0.76^abc^	0.48^cd^	0.49^bcd^	41.24^bc^	41.73^c^	52.15^ab^	52.43^ab^	45.45^bc^	45.59^bc^
ICM_4_	0.35^f^	0.38^cd^	0.73^bcd^	0.74^bc^	0.46^d^	0.47^cd^	39.55^cd^	41.00^cd^	50.26^abc^	50.52^abc^	45.16^bc^	45.29^bc^
ICM_5_	0.42^c^	0.41^b^	0.77^ab^	0.77^ab^	0.52^bc^	0.53^b^	43.81^ab^	44.21^b^	52.06^ab^	52.72^ab^	44.93^bc^	45.54^bc^
ICM_6_	0.38^de^	0.40^bc^	0.73^bcd^	0.74^abc^	0.49^cd^	0.50^bc^	40.44^cd^	41.99^c^	49.04^bc^	49.61^bc^	44.57^cd^	45.12^bc^
ICM_7_	0.51^a^	0.48^a^	0.77^a^	0.78^a^	0.58^a^	0.59^a^	45.64^a^	46.14^a^	52.86^a^	53.07^a^	48.19^a^	48.49^a^
ICM_8_	0.48^b^	0.46^a^	0.75^abc^	0.76^abc^	0.56^ab^	0.57^a^	43.68^ab^	44.18^b^	49.92^abc^	50.46^abc^	46.68^ab^	46.96^ab^
ICM_9_	0.31^g^	0.33^e^	0.70^d^	0.70^d^	0.45^d^	0.46d	33.44^f^	35.43^e^	45.30^d^	45.70^d^	43.06^d^	43.92^c^
SEm ±	0.008	0.009	0.014	0.013	0.014	0.013	0.93	0.62	1.11	1.08	0.60	0.86
LSD (0.05)	0.023	0.027	0.042	0.039	0.042	0.040	2.80	1.86	3.33	3.25	1.79	2.57
*Cropping systems (CS)*												
M–W	0.34^b^	0.39^b^	0.73^b^	0.73^b^	0.49	0.49	38.50^b^	39.98^b^	47.74^b^	48.11^b^	42.96^b^	43.38^b^
M + B–W	0.45^a^	0.41^a^	0.75^a^	0.76^a^	0.50	0.52	41.92^a^	42.57^a^	51.68^a^	52.14^a^	46.95^a^	47.29^a^
SEm ±	0.002	0.003	0.004	0.002	0.008	0.007	0.34	0.28	0.16	0.19	0.40	0.26
LSD (0.05)	0.006	0.009	0.011	0.007	NS	NS	1.00	0.82	0.47	0.55	1.17	0.76
ICM × CS	S	S	S	S	NS	NS	S	S	S	S	S	S

For detailed description of ICM, refer Table 2; maize–wheat cropping system (M–W); maize + blackgram–wheat cropping system (M + B–W).

^*a*^Values with different superscript letters in a column are significantly (*p* < 0.05) different.

### Chlorophyll content

The chlorophyll content (SPAD values) ranged from 33.4 to 53.1 across the ICM modules and at different intervals (Table 4). Chlorophyll content attained peak values at 60 DAS and later gradually decreased to its lowest at harvest. The SPAD values were similar between 100% RDF- and 75% RDF + NPK-bf + AMF-applied modules, except for OA-based ICM_9_ that recorded significantly (*p* < 0.05) lower SPAD than both 100 and 75% RDF-applied modules. CA-based ICM_7_ recorded SPAD values of 46.1, 53.1, and 48.5 at 30, 60, and 90 DAS, respectively, which were significantly (*p* < 0.05) higher than those recorded for CT- and OA-based modules. Over the entire crop duration, the association of blackgram as an intercrop improved the maize leaf chlorophyll content significantly (*p* < 0.05) over sole maize, where the increase was ∼7.1, 7.7, and 8.4% for 30, 60, and 90 DAS, respectively.

### Integrated crop management and cropping system interaction effect for normalised difference vegetation index

The ICM × cropping system interaction effect was significant (*p* < 0.05) for maize NDVI (Table 5). At 30 DAS in the respective 2 years (2019 and 2020), the blackgram-intercropped maize under CT-, CA-, and OA-based modules had 2.7–29.3%, 2.4–25.4% and 13.9–20.6% higher NDVI than sole maize, respectively. Similarly, at 60 DAS blackgram-intercropped maize under CT-, CA- and OA-based modules had 1.4–5.4, 1.3–10.4, and 1.4–2.8% higher NDVI than sole maize, respectively. CA-based modules were superior to CT- and OA-based modules at all times (Table 5) under blackgram intercropping; the behaviour of CT- and OA-based ICM modules was alike under sole maize and blackgram intercropping.

**TABLE 5:** Integrated crop management (ICM) modules × cropping system (CS) interaction effects on NDVI values of maize at 30-days interval under maize^a^.

ICM × CS	NDVI-30 DAS (2019)	NDVI-30 DAS (2020)	NDVI-60 DAS (2019)	NDVI-60 DAS (2020)	NDVI-90 DAS (2019)	NDVI-90 DAS (2020)
ICM_1_ × CS_1_	0.31^j^	0.36^hi^	0.77^abc^	0.74^def^	0.50^cdefg^	0.46^ef^
ICM_1_ × CS_2_	0.43^def^	0.40^cde^	0.71^efgh^	0.76^bcd^	0.43^g^	0.50^cdef^
ICM_2_ × CS_1_	0.29^k^	0.36^ghi^	0.70^fgh^	0.71^gh^	0.45^fg^	0.46^def^
ICM_2_ × CS_2_	0.41^f^	0.37^fghi^	0.74^bcde^	0.74^def^	0.46^defg^	0.47^def^
ICM_3_ × CS_1_	0.35^hi^	0.39^defg^	0.74^cdef^	0.74^ef^	0.46^defg^	0.47^def^
ICM_3_ × CS_2_	0.44^d^	0.39^def^	0.77^ab^	0.78^ab^	0.51^bcdef^	0.51^cde^
ICM_4_ × CS_1_	0.31^j^	0.40^cde^	0.72^efgh^	0.73^fg^	0.47^defg^	0.49^def^
ICM_4_ × CS_2_	0.39^g^	0.37^fghi^	0.73^cdefg^	0.75^def^	0.45^efg^	0.46^def^
ICM_5_ × CS_1_	0.37^h^	0.41^cd^	0.75^abcd^	0.76^cde^	0.53^abcd^	0.53^bcd^
ICM_5_ × CS_2_	0.47^c^	0.42^c^	0.78^a^	0.78^abc^	0.52^bcde^	0.53^bcd^
ICM_6_ × CS_1_	0.34^i^	0.38^efgh^	0.69^h^	0.70^hi^	0.47^defg^	0.48^def^
ICM_6_ × CS_2_	0.42^ef^	0.41^cd^	0.77^ab^	0.78^ab^	0.51^bcdef^	0.52^bcde^
ICM_7_ × CS_1_	0.44^de^	0.47^ab^	0.77^abc^	0.77^abc^	0.57^abc^	0.58^ab^
ICM_7_ × CS_2_	0.59^a^	0.49^a^	0.78^a^	0.79^a^	0.59^a^	0.60^a^
ICM_8_ × CS_1_	0.41^f^	0.46^b^	0.72^defgh^	0.73^fg^	0.55^abc^	0.56^abc^
ICM_8_ × CS_2_	0.54^b^	0.46^ab^	0.78^a^	0.79^a^	0.57^ab^	0.58^ab^
ICM_9_ × CS_1_	0.27^k^	0.31^j^	0.69^h^	0.69^i^	0.43^g^	0.43^f^
ICM_9_ × CS_2_	0.34^i^	0.36^i^	0.70^gh^	0.71^h^	0.47^defg^	0.48^def^
CS at same level of ICM SEm ±	0.01	0.01	0.01	0.01	0.02	0.02
LSD (0.05)	0.02	0.03	0.03	0.02	NS	NS
ICM at same or different levels of CS SEm ±	0.01	0.01	0.02	0.01	0.02	0.02
LSD (0.05)	0.03	0.03	0.05	0.04	NS	NS

For detailed description of ICM, refer Table 2; CS_1_-maize–wheat cropping system; CS_2_-maize + blackgram–wheat cropping system.

^*a*^Values with different superscript letters in a column are significantly (*p* < 0.05) different.

### Integrated crop management and cropping system interaction effect for soil plant analysis development

The ICM × cropping system interaction effect was significant (*p* < 0.05) for the chlorophyll content (SPAD) at all measurement times (Table 6). During 30 DAS, maize grown with blackgram intercropping under CT-, CA- and OA-based modules in the 2 years showed 2.1–13.9%, 1.4–11.0%, and 12.2–22.3% higher SPAD than sole maize, respectively. Similarly at 60 DAS, blackgram-intercropped maize under CT-, CA-, and OA-based ICM modules in the 2 years had 1.3–11.8, 6.6–11.0, and 12.2–13.5% higher SPAD than sole maize, respectively; the corresponding increase in SPAD values for 90 DAS was 1.1–12.2, 9.8–18.2, and 17.5–19.1% (Table 6). The highest SPAD value was found in blackgram-intercropped maize under ICM_7_ (55.6) at 60 DAS, and the lowest SPAD value was recorded in sole maize under ICM_9_ (29.2) at 30 DAS.

**TABLE 6:** Integrated crop management (ICM) modules × cropping system (CS) interaction effects on SPAD values of maize at 30-days interval DAS under maize^a^.

ICM × CS	SPAD-30 DAS (2019)	SPAD-30 DAS (2020)	SPAD-60 DAS (2019)	SPAD-60 DAS (2020)	SPAD-90 DAS (2019)	SPAD-90 DAS (2020)
ICM_1_ × CS_1_	35.2^ij^	38.2^f^	45.1^h^	45.5^g^	42.8^efg^	43.1^ghij^
ICM_1_ × CS_2_	40.9^efg^	40.9^de^	51.1^bcd^	51.6^bc^	44.5^cdef^	44.9^fgh^
ICM_2_ × CS_1_	34.2^j^	36.8^f^	46.7^g^	46.9^g^	41.9^fgh^	42.2^ij^
ICM_2_ × CS_2_	37.9^hi^	37.6^f^	48.8^ef^	49.3^def^	43.8^defg^	44.1^fghi^
ICM_3_ × CS_1_	39.8^fgh^	39.2^ef^	51.8^bc^	52.4^b^	42.5^efg^	42.7^hij^
ICM_3_ × CS_2_	42.7^cdef^	44.3^bc^	52.5^b^	52.4^b^	48.4^ab^	48.5^bc^
ICM_4_ × CS_1_	39.6^gh^	40.7^de^	48.7^f^	48.9^f^	44.9^bcdef^	45.0^fg^
ICM_4_ × CS_2_	39.4^gh^	41.3^de^	51.8^bc^	52.2^bc^	45.4^bcdef^	45.6^ef^
ICM_5_ × CS_1_	43.5^bcde^	42.4^cd^	50.2^de^	50.9^bcd^	40.4^gh^	41.0^jk^
ICM_5_ × CS_2_	44.1^abcd^	46.1^ab^	54.0^a^	54.5^a^	49.5^a^	50.1^ab^
ICM_6_ × CS_1_	39.1^gh^	41.0^de^	46.2^gh^	46.8^g^	41.9^fgh^	42.5^ij^
ICM_6_ × CS_2_	41.7^cdefg^	43.0^cd^	51.9^bc^	52.4^b^	47.2^abcd^	47.7^cde^
ICM_7_ × CS_1_	44.6^abc^	44.0^bc^	50.6^cd^	50.5^cde^	45.7^bcde^	46.0^def^
ICM_7_ × CS_2_	46.7^a^	48.3^a^	55.1^a^	55.6^a^	50.7^a^	51.0^a^
ICM_8_ × CS_1_	41.1^defg^	44.5^bc^	48.2^f^	48.7^f^	48.0^abc^	48.3^bcd^
ICM_8_ × CS_2_	46.2^ab^	43.9^bc^	51.6^bc^	52.2^b^	45.4^bcdef^	45.6^ef^
ICM_9_ × CS_1_	29.2^k^	33.1^g^	42.2^i^	42.4^h^	38.5^h^	39.7^k^
ICM_9_ × CS_2_	37.6^hi^	37.7^f^	48.4^f^	49.0^ef^	47.6^abc^	48.1^bcd^
CS at same level of ICM SEm ±	1.01	0.83	0.48	0.56	1.19	0.77
LSD (0.05)	3.01	2.47	1.42	1.65	3.52	2.28
ICM at same or different level of CS SEm ±	1.18	0.85	1.16	1.15	1.03	1.01
LSD (0.05)	3.52	2.55	3.48	3.45	3.07	3.03

For detailed description of ICM, refer Table 2; CS_1_-maize–wheat cropping system; CS_2_-maize + blackgram–wheat cropping system.

^*a*^Values with different superscript letters in a column are significantly (*p* < 0.05) different.

### Days taken to different phenological stages

**FIGURE 2:**
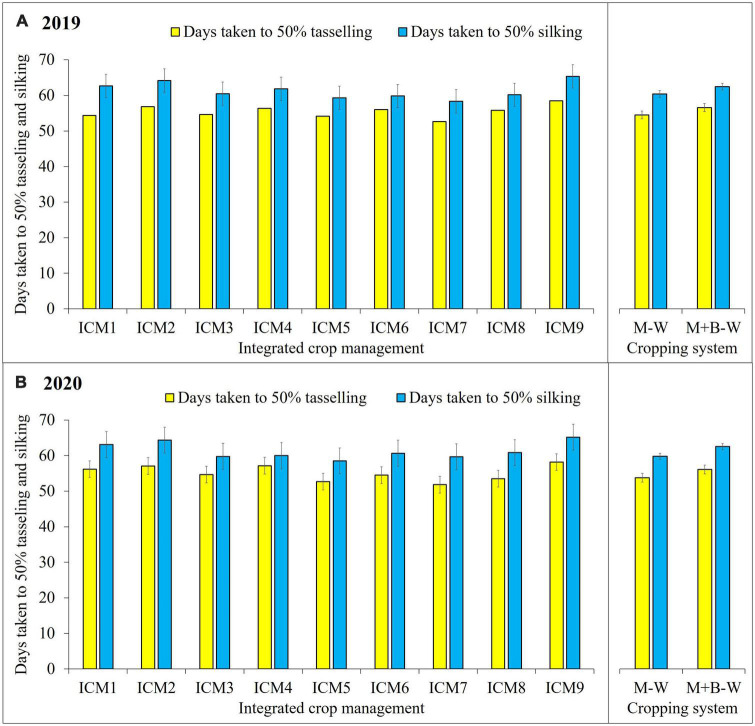
Effect of different ICM modules and cropping system on days taken to phenological stages of maize 2019 **(A)** and 2020 **(B)**. The LSD_0_._05_ is indicated by a bar above each column, and any treatment difference beyond the range of bar within each year is significantly different.

For attaining 50% tasselling, on an average 51.8–56, 54.7–57.2, and 58.2–58.5 days were taken by sole maize grown under CA-, CT-, and OA-based ICM modules, respectively (Figure 2). The maize crop took 58.5–60.8, 60.0–64.3, and 65.2–65.3 days to achieve 50% silking under CA-, CT-, and OA-based ICM modules, respectively. Here, it is interesting to summarise that blackgram intercropping prolonged the vegetative growth of maize and, thus, postponed the occurrence of 50% tasselling and silking by about 2.1–3.6 and 1.9–2.7 days, respectively, compared to sole maize cropping, which may help in curtailing the effect of early thermal heat stress in summer months in semiarid climates, besides enhancing the photosynthetic efficiency of the intercropped maize crop.

### Dry matter partitioning

**FIGURE 3:**
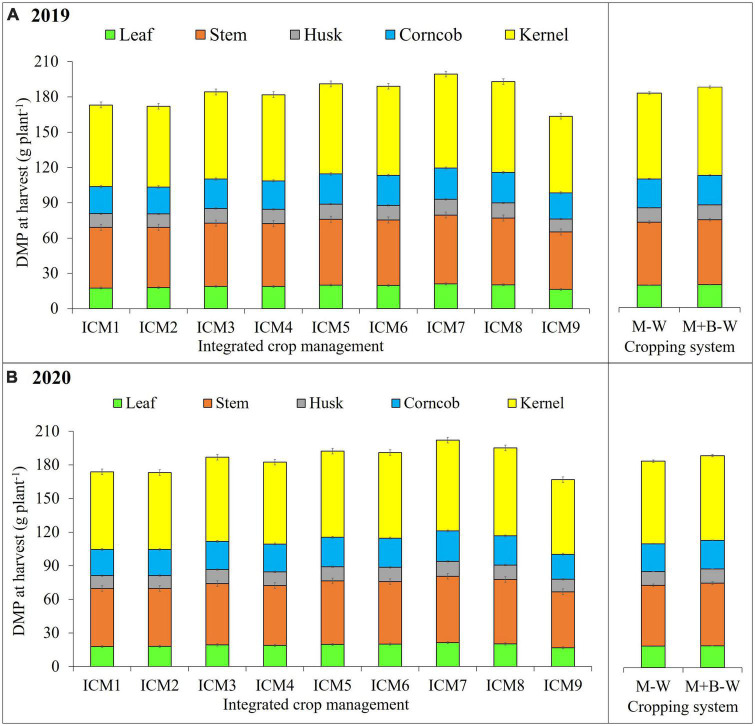
Effect of different ICM modules and cropping system on dry matter partitioning (DMP) of maize at harvest 2019 **(A)** and 2020 **(B)**. The LSD_0_._05_ is indicated by a bar above each column, and any treatment difference beyond the range of bar within each year is significantly different.

Leaf dry weight (LDW) and stem dry weight (SDW) ranged from 18.8 to 26.9 and 51.7 to 60.1 g plant^–1^. Both LDW and SDW were greater in CA-based ICM modules (Table 7). The OA-based ICM_9_ had the least LDW and SDW (Table 7). At harvest, the DMP among important plant parts as leaf, stem and kernels ranged between 16.4–21.4, 48.4–59.2, and 65.3–81 g plant^–1^, respectively (Figure 3). Kernel weight per plant was significantly (*p* < 0.05) higher under CA-based ICM modules, especially in ICM_7_ and ICM_8_. Dry matter accumulation (DMA) per plant forms the basis for DMP after flowering. The sum of DMP toward leaf, stem, husk, corncob and kernel was taken as DMA per plant. Thus, DMA was significantly (*p* < 0.05) higher under CA-based ICM modules (ICM_5_ to ICM_8_) (Figure 3); likewise, the DMP toward different plant parts was also higher under CA. And, the CA-based ICM_7_ had the highest DMP of 79.8 and 81 g kernel per plant during 2019 and 2020, respectively; while among the CT-based modules, ICM_3_ had significantly (*p* < 0.05) higher DMP to kernel 74.1 and 75.2 g per plant during 2019 and 2020, respectively. The least kernel DMP was observed in ICM_9_, i.e., 65.3 and 66.6 g per plant during 2019 and 2020, respectively. Similar trend was observed in DMP of leaf and stem, in the 2 years. Blackgram intercropping significantly (*p* < 0.05) improved the leaf (1.7–3.0%), stem (2.8–3.0%), and kernel (2.5%) weight per plant in 2019 and 2020, respectively (Figure 3).

**TABLE 7:** Effect of ICM modules and cropping system on dry matter accumulation and leaf growth parameters of maize at flowering stage^a^.

Treatments	Dry matter accumulation (g plant^–1^)				Leaf weight ratio		Specific leaf area (cm^2^ mg^–1^)		Specific leaf weight (mg cm^–2^)	
				
	Leaf dry wt. (LDW)		Stem dry wt. (SDW)							
	2019	2020	2019	2020	2019	2020	2019	2020	2019	2020
*Integrated crop management (ICM)*										
ICM_1_	20.8^f^	21.9^cd^	53.8^f^	54.9^cd^	0.279^f^	0.285^cd^	0.26	0.25	3.82	3.95
ICM_2_	21.1^ef^	21.9^cd^	54.1^ef^	55.0^cd^	0.280^ef^	0.285^cd^	0.25	0.24	3.97	4.15
ICM_3_	22.7^de^	23.3^bc^	55.8^de^	56.4^bc^	0.289^de^	0.292^bc^	0.27	0.27	3.72	3.78
ICM_4_	22.3^def^	22.7^c^	55.3^def^	55.8^c^	0.286^def^	0.289^c^	0.27	0.27	3.74	3.75
ICM_5_	24.6^bc^	24.9^ab^	57.7^bc^	58.0^ab^	0.299^bc^	0.300^ab^	0.26	0.26	3.86	3.85
ICM_6_	23.9^cd^	25.0^ab^	57.0^cd^	58.1^ab^	0.296^cd^	0.301^ab^	0.25	0.25	3.99	4.10
ICM_7_	26.7^a^	26.9^a^	59.9^a^	60.1^a^	0.308^a^	0.309^a^	0.25	0.25	4.01	3.95
ICM_8_	26.1^ab^	26.5^a^	59.3^ab^	59.7^a^	0.306^ab^	0.308^a^	0.25	0.25	4.05	4.03
ICM_9_	18.8^g^	20.1^d^	51.7^g^	53.1^d^	0.266^g^	0.275^d^	0.26	0.24	3.93	4.15
SEm ±	0.6	0.7	0.6	0.7	0.003	0.004	0.01	0.01	0.13	0.16
LSD (0.05)	1.7	2.1	1.8	2.2	0.010	0.011	NS	NS	NS	NS
*Cropping systems (CS)*										
M–W	22.1^b^	22.9^b^	55.2^b^	55.9^b^	0.285^b^	0.289^b^	0.26	0.25	3.88	3.96
M + B–W	23.9^a^	24.6^a^	57.0^a^	57.6^a^	0.295^a^	0.298^a^	0.26	0.25	3.92	3.98
SEm ±	0.2	0.1	0.2	0.1	0.001	0.001	0.002	0.001	0.03	0.02
LSD (0.05)	0.6	0.3	0.7	0.4	0.003	0.002	NS	NS	NS	NS
ICM × CS	NS	NS	NS	NS	NS	NS	NS	NS	NS	NS

For detailed description of ICM, refer Table 2; maize–wheat cropping system (M–W); maize + blackgram–wheat cropping system (M + B–W).

^*a*^Values with different superscript letters in a column are significantly (*p* < 0.05) different.

### Leaf characteristics

Leaf weight ratio (LWR) indicates the ratio between LDW and whole plant dry weight. The higher LWR denotes a higher proportion of leaf in plant dry weight and hence greater photosynthetic area of plant. Crop establishment pattern and nutrient management option stood at par, excluding OA-based ICM_9_. CA-based ICM modules resulted in significantly (*p* < 0.05) higher LWR than CT and OA (Table 7). Intercropped maize recorded significantly higher (3.0–3.4%) LWR than sole maize. Both ICM and CS did not exert any significant (*p* < 0.05) effect on specific leaf area (SLA) and specific leaf weight (SLW) measured during the flowering stage. The interaction effect of ICM and CS on LWR, SLA and SLW was non-significant during both years (Table 7).

### Leaf area index and leaf area ratio

Leaf area index (LAI) range was 1.84–2.23, 3.42–4.88, and 2.68–3.67 at 30, 60, and 90 DAS, respectively (Table 8). Similarly, leaf area ratio (LAR) range was 94.0–106.5, 66.5–78.6, and 24.6–28.9 cm^2^ g^–1^ at 30, 60, and 90 DAS, respectively. Both ICM and CS had a significant (*p* < 0.05) effect on LAI and LAR in both years. CA-based ICM_7_ caused significantly (*p* < 0.05) greater LAI (2.19, 4.83 and 3.63) at 30, 60, and 90 DAS, which was ∼9.1, 18.8, and 14.3% higher than CT-FB-based ICM modules and ∼14.4, 28.7, and 25.9% higher than OA ICM_9_ module, respectively. The CA-based ICM modules exhibited significantly (*p* < 0.05) lower LAR at 30 DAS, while the OA-based ICM_9_ had significantly (*p* < 0.05) lower LAR at 60 and 90 DAS (Table 8). Sole maize had 104.8 cm^2^ leaf area g^–1^ of LDW, which was higher at 30 DAS, while intercropped maize had 75.3 and 28.6 cm^2^ leaf area g^–1^ of LDW, which was significantly (*p* < 0.05) higher than sole maize during 60 and 90 DAS, respectively. For both LAI and LAR, ICM modules had a significant (*p* < 0.05) interaction with CS up to 60 DAS, but it was non-significant at 90 DAS. Blackgram-intercropped maize had 3.2, 4.6, and 7.2% greater LAI than sole maize at 30 DAS while on interaction with ICM, under CT, CA and OA, respectively. Similarly, at 60 DAS, blackgram-intercropped maize had 5.2, 6.6, and 12.5% higher LAI than sole maize under CT, CA, and OA, respectively. At 30 DAS, the LAR of OA- and CT-based ICM modules was significantly (*p* < 0.05) higher than CA-based ICM modules. But at 60 DAS, the LAR of CA- PRB-, CA-FB- and CT-RB-based ICM modules stood at par.

**TABLE 8:** Effect of ICM modules and cropping system on leaf area index (LAI) and leaf area ratio (LAR) of maize at 30-days interval^a^.

Treatments	LAI						LAR (cm^2^ g^–1^)					
	30 DAS		60 DAS		90 DAS		30 DAS		60 DAS		90 DAS	
	2019	2020	2019	2020	2019	2020	2019	2020	2019	2020	2019	2020
*Integrated crop management (ICM)*												
ICM_1_	1.95^c^	2.04^bc^	3.89^d^	3.96^e^	3.11^c^	3.12^d^	106.4^ab^	107.4^a^	73.0^abc^	72.3^abc^	27.6^bc^	27.5^bc^
ICM_2_	1.94^c^	2.02^c^	3.79^d^	3.78^e^	2.93^d^	2.98^d^	109.9^a^	111.6^a^	70.6^bc^	68.8^bc^	26.2^cd^	26.0^c^
ICM_3_	2.00^bc^	2.10^b^	4.36^c^	4.41^cd^	3.41^ab^	3.48^bc^	106.0^ab^	104.2^abc^	78.0^a^	77.7^a^	29.1^ab^	29.4^a^
ICM_4_	1.97^c^	2.06^bc^	4.26^c^	4.32^d^	3.39^b^	3.42^bc^	106.4^ab^	106.6^ab^	77.2^a^	77.2^a^	29.5^a^	29.6^a^
ICM_5_	2.06^b^	2.11^b^	4.56^c^	4.63^bc^	3.45^ab^	3.52^ab^	100.0^bc^	99.2^bc^	77.6^a^	78.1^a^	29.1^b^	29.5^a^
ICM_6_	1.99^bc^	2.08^bc^	4.29^c^	4.38^d^	3.29^bc^	3.34^c^	104.4^ab^	105.1^ab^	74.3^ab^	73.7^ab^	28.2^ab^	28.3^ab^
ICM_7_	2.16^a^	2.23^a^	4.78^a^	4.88^a^	3.59^a^	3.67^a^	94.2^c^	97.1^c^	77.5^a^	78.6^a^	28.8^ab^	29.5^a^
ICM_8_	2.06^b^	2.18^a^	4.61^ab^	4.70^ab^	3.43^ab^	3.52^ab^	94.0^c^	97.2^c^	75.6^ab^	76.4^a^	28.9^ab^	29.2^ab^
ICM_9_	1.84^d^	1.91^d^	3.42^e^	3.47^f^	2.68^e^	2.70^e^	106.5^ab^	108.1^a^	67.9^c^	66.5^c^	24.6^d^	25.8^c^
SEm ±	0.03	0.02	0.06	0.08	0.06	0.05	2.8	2.5	1.9	2.2	0.6	0.6
LSD (0.05)	0.09	0.06	0.19	0.24	0.18	0.16	8.3	7.5	5.6	6.5	1.7	1.8
*Cropping systems (CS)*												
M–W	1.95^b^	2.01^b^	4.08^b^	4.15^b^	3.13^b^	3.19^b^	104.7^a^	104.9^a^	73.8^b^	73.6^b^	27.5^b^	27.9^b^
M + B–W	2.05^a^	2.16^a^	4.36^a^	4.42^a^	3.38^a^	3.42^a^	101.5^b^	103.2^b^	75.4^a^	75.1^a^	28.5^a^	28.8^a^
SEm ±	0.01	0.01	0.01	0.01	0.01	0.01	0.6	0.5	0.4	0.3	0.1	0.1
LSD (0.05)	0.02	0.02	0.04	0.03	0.03	0.03	1.7	1.5	1.1	0.9	0.3	0.4
ICM × CS	S	S	S	S	NS	NS	S	S	S	S	S	NS

For detailed description of ICM, refer Table 2; maize–wheat cropping system (M–W); maize + blackgram–wheat cropping system (M + B–W).

^*a*^Values with different superscript letters in a column are significantly (*p* < 0.05) different.

The ICM and cropping system interaction effect was significant (*p* < 0.05) for maize LAI and LAR only during 30 and 60 DAS. Irrespective of tillage, crop establishment methods and nutrient/water management options, at 30 and 60 DAS, the maize under blackgram-intercropped ICM modules recorded significantly higher LAI than sole maize during both years. While at 30 DAS, the blackgram-intercropped maize recorded significantly lower LAR than sole maize, except CA-PRB (ICM_7_ and ICM_8_), where the maize + blackgram intercropping had significantly higher LAR. At 60 DAS, maize crop under ICM modules with blackgram intercropping had higher LAR than sole maize. At 90 DAS, the ICM and cropping system interaction was non-significant for LAI and LAR, during both years.

### Crop productivity

#### Combined maize equivalent grain yield

**FIGURE 4:**
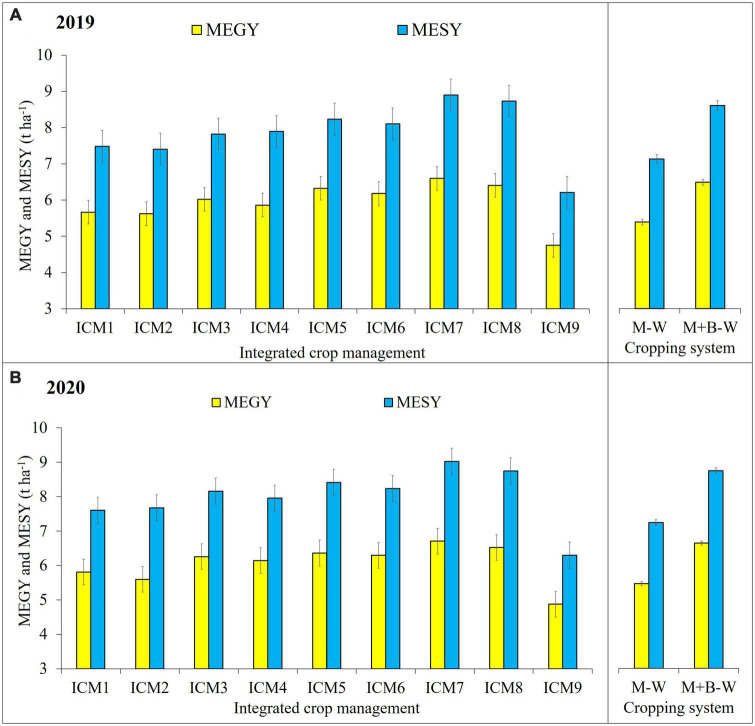
Effect of different ICM modules and cropping system on maize equivalent grain yield (MEGY) and maize equivalent stover yield (MESY) 2019 **(A)** and 2020 **(B)**. The LSD_0_._05_ is denoted by a bar above each column, and any treatment beyond the range of bar within each year is significantly different.

Combined MEGY was significantly (*p* < 0.05) influenced by both ICM modules and cropping systems (Figure 4). The MEGY was highest in ICM_7_ (6.6 and 6.71 t ha^–1^), while the remaining ICM modules followed the decreasing order as ICM_8_ > ICM_5_ > ICM_6_ > ICM_3_ > ICM_4_ > ICM_1_ > ICM_2_ > ICM_9_. Nutrient management options like 100% RDF and 75% RDF + NPK-*bf* + AM fungi stood on par with each other. Under various crop establishment techniques, MEGY was significantly (*p* < 0.05) higher in CA-PRB-induced ICM modules followed by CA- FB-, CT-RB- and CT-FB-based ICM modules. It is vividly evident from Figure 4 that CA-based ICM modules were superior to CT- and OA-based ICM modules during both years of study. The MEGY of CA-based ICM_7_ was 13.8 and 27.9% higher than CT-FB- and OA-based ICM modules. The MEGY of blackgram-intercropped maize was 6.49 and 6.66 t ha^–1^, which was higher by 16.9 and 17.9% over sole maize in 2019 and 2020, respectively. ICM and CS had a significant (*p* < 0.05) interaction effect on MEGY only during 2020. The CA-PRB ICM_7_ module in combination with blackgram intercropping recorded highest MEGY (7.32 t ha^–1^), which was 16.7% higher than sole maize under ICM_7_ module.

#### Combined stover yield (maize equivalent stover yield)

The ICM modules and CS had a significant effect on MESY, whose general trend was similar to that of MEGY (Figure 4). The MESY under different nutrient management options were on par, except for the OA-based ICM_9_ (where 15 t ha^–1^ FYM was applied instead of RDF), which recorded significantly (*p* < 0.05) lower stover yield in 2019 and 2020, respectively (6.21 and 6.3 t ha^–1^). The CA-based ICM_7_ recorded higher MESY (8.9 and 9.02 t ha^–1^) than CT. Also, the ICM modules with RB/PRB land configuration proved superior to FB-based ICM modules under CT, CA and OA. Blackgram-intercropped maize produced 8.6 and 8.77 t ha^–1^ of MESY against MESY of 7.13 and 7.26 t ha^–1^ obtained from sole maize during 2019 and 2020, respectively. During 2020, ICM and CS had a significant (*p* < 0.05) interaction effect on MESY. The blackgram-intercropped ICM_7_ module recorded highest MESY (9.98 t ha^–1^), which was 21.6% higher than sole maize under ICM_7_ module.

### Multivariate and principal component analysis

**FIGURE 5:**
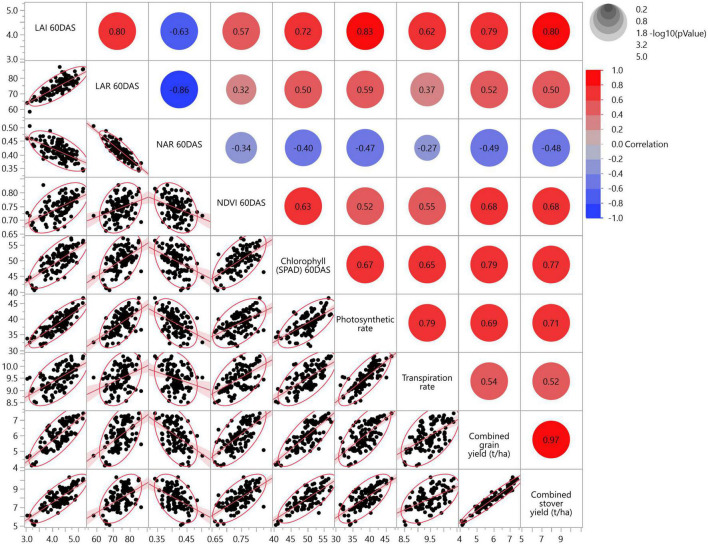
Multivariate analysis showing correlation among various crop physiological (LAI, LAR, NAR, NDVI and SPAD at 60 DAS) and photosynthetic characteristics (flowering stage, 60 DAS), MEGY and MESY in maize from two cropping seasons (2019 and 2020). The lower triangle shows scatter plot matrix with line fit, and the upper triangle shows the significant circles with correlation coefficient (*p* < 0.05) (2 years’ pooled data).

The MVA and PCA were performed to understand the strength and degree of correlation among physiological parameters, crop growth, health and productivity (MEGY and MESY). In general, all observed parameters were found positively correlated with MESY and MEGY, except for the negative correlation with the NAR (Figure 5). Positive correlations included those of combined grain yield (MEGY), with chlorophyll (SPAD) at 60 DAS (*r* = 0.79), LAI (*r* = 0.79), P_n_ (*r* = 0.69), NDVI (*r* = 0.68), T_r_ (*r* = 0.54). At 60 DAS, a strong negative correlation was observed in NAR, with LAR (*r* = –0.86), LAI (*r* = –0.63). A strong positive correlation was found between MEGY and MESY with a correlation coefficient of 0.97. Similarly, P_n_ was positively correlated with several parameters, including SPAD (*r* = 0.67), T_r_ (*r* = 0.79), and LAI (*r* = 0.83).

**FIGURE 6:**
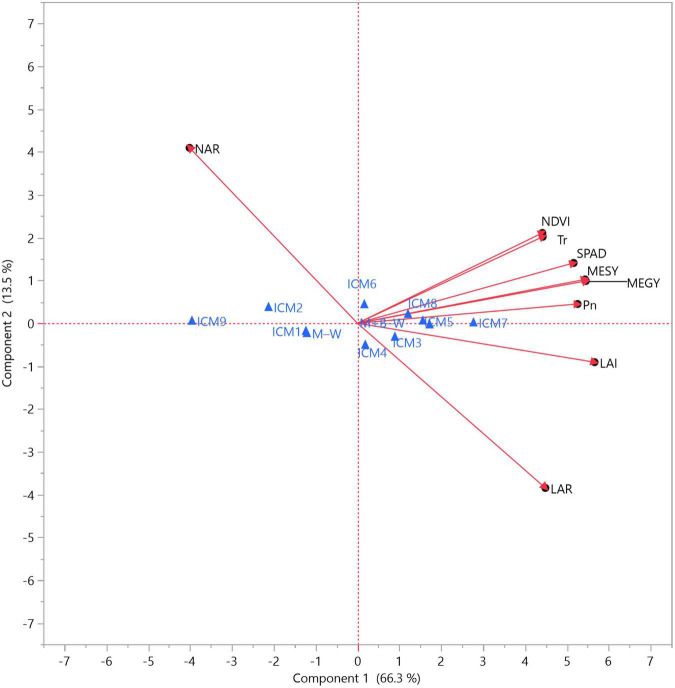
Principal component analysis (PCA) biplots on impact of ICM and CS on various crop physiological (LAI, LAR, NAR, NDVI, and SPAD at 60 DAS) and photosynthetic characteristics (flowering stage, 60 DAS), MEGY and MESY in maize from two cropping seasons (2019 and 2020). Blue triangle indicates main- and subplot treatments, i.e., CT (ICM_1_–ICM_4_), CA (ICM_5_–ICM_8_), and OA (ICM_9_); maize–wheat cropping system (M–W); maize + blackgram–wheat cropping system (M + B–W).

Moreover, PCA illustrates the significant effect of individual treatments (ICM and CS) and among various observed parameters (Figure 6). The PCA shows the superiority of blackgram-intercropped maize over sole maize. Similarly, all CA-based modules (ICM_5_–ICM_8_) and CT-RB modules (ICM_3_ and ICM_4_) performed more or less similar in improving the maize productivity by improving all physiological, crop vigour and growth parameters (Figure 6). All physiological parameters as P_n_, T_r_, SPAD and NDVI were closely related to MEGY and MESY, except LAR and NAR which were found to be far from being related.

## Discussion

### Photosynthetic characteristics

Grain formation and crop productivity immensely depend on the photosynthetic characteristics of the crop (Long et al., 2006; Dass and Chandra, 2013; Kumari et al., 2017; Choudhary et al., 2022). In our study, we found that ICM practices significantly influenced the maize photosynthetic characteristics. Maize under CA-based ICM modules showed significant (*p* < 0.05) improvement in net photosynthetic rate (P_n_), transpiration rate (T_r_), stomatal conductance (G_s_) and transpiration efficiency (T_E_); the magnitude of improvement ranged from 9.0 to 9.2% for P_n_, 4.5 to 4.6% for T_r_, 6.0 to 6.1% for G_s_ and 4.6 to 4.9% for T_E_ over CT-based ICM modules, in 2019 and 2020, respectively. Blackgram intercropping had a positive influence on the maize crop and substantially improved all its photosynthetic characteristics as P_n_, T_r_, and T_E_ over sole maize by increasing LAI, LAR, and LWR (Li et al., 2019). Blackgram intercropping favours maize through (i) reducing N leaching to deeper soil layers (Whitmore and Schröder, 2007; Ding et al., 2021), (ii) symbiotic nitrogen fixation (Ibrahimi et al., 2017; Weil and Brady, 2017) and (iii) smothering and suppressing the weed growth (Saudy, 2015; Choudhary and Choudhury, 2018; Choudhary et al., 2020; Gu et al., 2021), which ultimately improves soil N content. Many studies have shown that increased soil N enhances photosynthetic properties and NAR of maize (Zhang et al., 2014; Guo Y. et al., 2021; Guo F. et al., 2021). Under normal ecosystem, legumes can fix up to 28.0–84.1 kg N ha^–1^ (Kebede, 2021), while the amounts of nitrogen transfer from legume intercrop to companion crop vary between 21.6 and 50.9 kg N ha^–1^ (Mallarino et al., 1990; Fustec et al., 2010; Thilakarathna et al., 2016). In most cases, the ICM modules under the 100% RDF nutrition management option performed similarly to the 75% RDF + NPK-*bf* + AMF, demonstrating that the remaining 25% of nutrient requirements could be met through the synergistic action of microbial consortia and AMF (Mishra et al., 2021; Singh et al., 2021, 2022a; Khan, 2022). Similarly, the combined action of NPK-*bf* and AMF on P solubilisation and P mobilisation might have increased P bioavailability and shoot and root growth (Suri et al., 2011a,b; Kumar et al., 2015). The enhancement in root growth and root exploratory area due to mycorrhizal mycelia growth under AMF-applied plots may further lead to enhanced nutrient acquisition, plant growth and photosynthetic characteristics of the crop (Kumar et al., 2021, 2022; Harish et al., 2022). Similarly, the ZT system under CA-based ICM modules leads to improved nutrient bioavailability and soil fertility, soil structure, moisture conservation, microclimate modulation and more favourable soil microbiome under the harsh summer season of semiarid IGPR coinciding with an early vegetative phase of maize. All these circumstances resulted in improved plant growth and photosynthetic characteristics in maize under CA-based ICM modules (Choudhary et al., 2018, 2020; Varatharajan et al., 2019a,b; Singh et al., 2020, 2021, 2022a,2022b; Kumar et al., 2021, 2022).

### Crop growth and physiological parameters

In the current study, the ICM combining different tillage and crop establishment techniques, nutrient management scenarios and crop residue retention had a significant (*p* < 0.05) impact on crop growth and health, as measured by NDVI and SPAD. The higher is the NDVI, the better is the plant water conditions and, hence, greener and thicker/denser is the crop canopy (Varatharajan et al., 2019a; Harish et al., 2021). That is why the sole maize or maize + blackgram intercropping in CA-based residue retained PRB plots with 100% RDF application (ICM_5_, ICM_7_) was greener, denser and with less water stress than OA-based ICM_9_ or CT-based FB plots with 75% RDF + NPK-*bf* + AMF allocation (ICM_2_, ICM_4_). Likewise, it is evident from our research that CA with PRB and 100% RDF application significantly (*p* < 0.05) improved the chlorophyll content (SPAD greenness) of maize plants than CT/OA with FB and 75% RDF + NPK-*bf* + AMF (Saudy, 2014). This was because of the improved nutrient bioavailability, lower water stress, superior soil microbial activity and favourable soil microclimate for nutrient uptake (Singh et al., 2021, 2022a; Harish et al., 2022). Further, the nutrient released from slow decomposing residues retained on CA plots might have positively impacted crop health over the entire crop duration (Jayaraman et al., 2021). ICM and cropping system interaction for NDVI and SPAD also reiterates the above facts. At the same time, the improved degradation of residue retained and the subsequent nutrients release along with improved soil microbial activity under CA might increase the available nutrients in the soil and ensure nutrient supply to maize as and when required (Sarkar et al., 2020; Jayaraman et al., 2021; Kumar et al., 2022). This might reduce the requirement for additional nutrient supplements from legume intercrop. Hence, the effect of legume intercropping was more prominent in CT/OA than CA, which denotes less available nutrients in CT/CA, which are supplemented through legume inclusion as intercrop.

Maize and blackgram intercropping caused significant (*p* < 0.05) improvement in LAI, LAR and LWR, while SLA and SLW did not change significantly (*p* < 0.05). Similarly, ICM exerted a significant (*p* < 0.05) improvement in DMP at the harvest stage (Figures 3A,B). Blackgram intercropping expressed its significant influence on DMA and translocation (leaf, stem, kernel) by improving maize photosynthesis and growth. In the current study, we found that maize under CA-based ICM plots had significantly higher DMA than CT and OA. Under CA, the higher DMA in leaves and stem before flowering might have helped in improving the photosynthates translocation from leaves and stem toward the kernel in post-anthesis and grain-filling stages due to enhanced N enrichment and bioavailability (Dhillon et al., 2018). Thus, increased kernel DMA suggests the improved photosynthates’ translocation efficiency from leaves and stem to sink (kernel) during grain filling. Similarly, blackgram intercropping increased the maize kernel DMP, as a result of N rhizodeposition from legume roots and improved soil nitrogen bioavailability (Fustec et al., 2010; Amanullah et al., 2021). The DMA under intercropping system also depends on the intercrop, its growth habit and competitive efficiency. In contrast to this, some researchers also reported contrasting results that intercropping negatively affected DMA and productivity of base crop (Huang et al., 2019, Arshad, 2021). Thus, the selection of the right intercrop components is very important, even though the spatial arrangement of crops may reduce competition to some extent (Dass and Sudhishri, 2010). Legume intercrop serves as an additional source of carbon input for better soil microbial activity and nutrient cycling (Choudhary et al., 2020; Singh et al., 2021, 2022a). Thus, CA and legume intercropping combinations may lead to improved soil fertility in the long run, which might contribute substantially toward the improved crop growth, DMA and DMP in the crops (Bantie, 2019). Hence, maize under CA-based ICM modules with blackgram intercropping had significantly (*p* < 0.05) higher leaf area per gram of plant dry weight (LAR) and leaf weight to plant dry weight (LWR), which denotes a higher proportion of leaf in whole plant dry weight. Eventually, this results in higher light interception, photosynthetic area and NAR, which in turn increases final crop productivity (Long et al., 2006). The role of crop growth and vigour in improving the photosynthetic characteristics and MEGY and MESY can be supported from our findings based on the correlation among LAI, LAR, NDVI, P_n_ and SPAD with MEGY and MESY, as well as multivariate (Figure 5) and PCA analysis (Figure 6).

### Crop productivity

Significant (*p* < 0.05) improvement in plant growth and photosynthetic characteristics, NDVI, SPAD and better DMP led to increase in the maize yield under intercropping system than the sole maize cultivation. The RB/PRB planting had an efficient and denser crop canopy with higher light capture and use efficiency than FB planting, which might have helped in yield enhancement. Likewise, crop residue retained under CA-PRB reduced evaporation losses which led to improved crop productivity and water-use efficiency (WUE) even with less amount of water applied (45 mm/irrigation) compared to FB (60 mm/irrigation). However, likely greater evaporation loss and higher competition from weeds resulting from less denser crop canopy under FB-based ICM modules eventually led to reduction in crop productivity and WUE. Besides, intercropped blackgram increases the photosynthetically active radiation (PAR) interception by utilising the transmitted radiation from maize crop by actively growing and shading the ground (Awal et al., 2006; Liu et al., 2017). Thus, the intercropping increases the MEGY of the system. In addition, atmospheric nitrogen fixation, nutrient exudates from legume roots under blackgram intercropping might have been beneficial in increasing the grain and stover yield of maize (Kakraliya et al., 2018; Pooniya et al., 2018). The intercropped maize has a substantial advantage over the sole maize cultivation in higher light interception from understorey blackgram due to the increased LAI and land covering (Rajpoot et al., 2018). This, in turn, increases light-use efficiency and crop productivity of intercropping system than sole crop (Kermah et al., 2017; Rajpoot et al., 2018). Additional intercrop yield further improves the land-use efficiency by improving the land equivalent ratio (Xu et al., 2020; Feng et al., 2021). By boosting the rhizospheric N and P cycling through microbial populations, the CA practices enhance soil available N and P (Singh et al., 2021, 2022a,2022b). Furthermore, under the maize–legume intercropping system, the legume component considerably influences the carbon, nitrogen and phosphorus metabolism in the soil, hence, improving nutrient bioavailability to both the base crop and the intercrop (Guo Y. et al., 2021; Singh et al., 2021, 2022a). Likewise, the crop residue retention and its decomposition under CA-based ICM modules might have improved the SOC, nutrient bioavailability, soil moisture retention, microclimate modulation and biological activity, hence all resulting in greater growth, photosynthesis and finally crop productivity (Varatharajan et al., 2019b; Choudhary et al., 2020; Singh et al., 2020, 2021, 2022a,b; Harish et al., 2022). Furthermore, the ZT system under CA-based ICM leads to favourable characteristics, such as reduced resource competition, root aeration and high fertiliser use efficiency, which might have resulted in higher grain output than the CT-based ICM modules (Harish et al., 2021, 2022). Lesser farm machinery trafficking, better root aeration and improved soil health parameters lead to improved shoot and root growth, which again might have contributed in enhancing the maize productivity in CA plots (Varatharajan et al., 2019b; Kumar et al., 2021, 2022). It is also noticed that there is lesser water stagnation after heavy rains vis-à-vis enhanced moisture retention during dry spells in maize under the CA system (Choudhary et al., 2018, 2020; Harish et al., 2021, 2022). In nutshell, all these favourable conditions under the CA-based modules led to overall superior physiological and photosynthetic characteristics with less environmental stress which collectively resulted in better crop productivity.

## Conclusion

The present study provides major insights into the positive impacts of the ICM modules and legume intervention on crop physiological and photosynthetic characteristics and productivity of sole and intercropped maize. Among the ICM modules, conservation agriculture (CA)-based ICM was superior to conventional tillage (CT)- and organic agriculture (OA)-based ICM for all the studied parameters, including overall productivity. In general, the CA-based ICM module ICM_7_ proved superior with significant improvements in photosynthetic rate, transpiration rate and NAR, crop vigour, DMP to the grain and finally maize productivity by 13.4–14.2% and 27.3–28.0% higher than the CT- and OA-based modules in 2019 and 2020, respectively. Nutrient management options like 100% RDF and 75% RDF + NPK-*bf* + AMF also stood on par with each other. Furthermore, blackgram intercropping in maize played a significant role in improving maize productivity over sole maize due to improved crop physiological and photosynthetic characteristics. Likewise, MEGY was significantly (*p* < 0.05) higher in CA-PRB-based ICM modules followed by CA-FB-, CT-RB- and CT-FB-based ICM modules. The MEGY of blackgram-intercropped maize was higher by 16.9–17.9% over sole maize in the current study. The MEGY followed the trend of ICM_7_ > ICM_8_ > ICM_5_ > ICM_6_ > ICM_3_ > ICM_4_ > ICM_1_ > ICM_2_ > ICM_9_. Overall, crop physiological and photosynthetic characteristics and maize productivity were found to be significantly higher in the CA-based ICM_7_ module with blackgram intercropping; hence, this technology package can be adopted widely to enhance the maize production in maize-growing semiarid regions of India and South Asia.

## Data availability statement

The original contributions presented in this study are included in the article/supplementary material, further inquiries can be directed to the corresponding author/s.

## Author contributions

TV: conceptualisation, methodology formulation and implementation, resource, review, and original draft preparation. AD: conceptualisation, project administration, methodology formulation and implementation, and review and editing. AC: conceptualisation, project administration, methodology formulation, and review and editing. SS and KS: results compilation and draft preparation. VP, SP, AS, PK, and TD: review of literature and basic analysis. GR: results compilation and draft preparation. MH: results compilation and draft preparation and editing. SD and RS: data collection and processing and original draft preparation. RR: review of literature and results compilation. KK: editing of original and revised versions of the manuscript. KSS: data collection and processing and original draft preparation. All authors contributed to the article and approved the submitted version.

## Abbreviations

ANOVA: analysis of variance
AMF: arbuscular mycorrhizal fungi
CA: conservation agriculture
CS: cropping system
CT: conventional tillage
DAP: diammonium phosphate
DAS: days after sowing
DMA: dry matter accumulation
DMP: dry matter partitioning
FB: flatbed
G_*s*_: stomatal conductance
ha: hectare
ICM: integrated crop management
IGPR: Indo-Gangetic plains region
IRGA: infrared gas analyser
K: potassium
MEBY: maize equivalent biological yield
MEGY: maize equivalent grain yield
MESY: maize equivalent stover yield
MVA: multivariate analysis
MWCS: maize–wheat cropping system
N: nitrogen
NAR: net assimilation rate
NDVI: normalised difference vegetation index
NPK-bf: NPK biofertiliser
LAI: leaf area index
LAR: leaf area ratio
LDW: leaf dry weight
LWR: leaf weight ratio
OA: organic agriculture
P: phosphorus
P_*n*_: net photosynthetic rate
PAR: photosynthetically active radiation
PCA: principal component analysis
PRB: permanent raised bed
RB: raised bed
RUE: resource-use efficiency
RDF: recommended dose of fertilizer
RWCS: rice–wheat cropping system
SDW: stem dry weight
SLA: specific leaf area
SOC: soil organic carbon
SLW: specific leaf weight
SPAD: soil plant analysis development
T_*r*_: leaf transpiration rate
t ha^–1^: tonnes ha^–1^
ZT: zero tillage.
